# Complete Mitochondrial Genome of King Threadfin, *Polydactylus macrochir* (Günther, 1867): Genome Characterization and Phylogenetic Analysis

**DOI:** 10.3390/genes16010088

**Published:** 2025-01-15

**Authors:** Jiufu Wen

**Affiliations:** 1 Key Laboratory of South China Sea Fishery Resources Exploitation and Utilization, Ministry of Agriculture and Rural Affairs, South China Sea Fisheries Research Institute, Chinese Academy of Fishery Sciences, Guangzhou 510300, China; nhswjf@163.com; 2 Hainan Engineering Research Center for Deep-Sea Aquaculture and Processing, Sanya 572018, China

**Keywords:** threadfin, Polynemidae, mitogenome

## Abstract

Background: *Polydactylus macrochir* (Günther; 1867) is a member of the family Polynemidae. The placement of Polynemidae among teleosts has varied over the years. Methods: Therefore, in this study, we sequenced the complete mitochondrial genome of *P. macrochir*, analyzed the characterization of the mitochondrial genome, and investigated the phylogenetic relationships of Polynemidae. Results: The length of the *P. macrochir* mitogenome was 16,738 bp, with a typical order. Nucleotide composition analysis showed that the *P. macrochir* mitogenome was AT-biased (54.15%), and the PCGs tended to use A and C rather than T and G at the third codon. All the PCGs started with the regular codon ATG, except for *cox1*, which started with GTG. The termination codon varied across the PCGs. It was shown that the ka/ks ratios of all the PCGs were less than one. Phylogenetic analysis, based on the maximum likelihood (ML) and Bayesian inference (BI) methods, indicated that eight threadfins formed a well-supported monophyletic cluster. Polynemidae and Sphyraenidae clustered together as a monophyletic group. According to TimeTree analyses, the most recent common ancestor (MRCA) of Polynemidae was traced back to about 52.81 million years ago (MYA), while six species within Polynemidae diverged from 11.70 MYA to 20.05 MYA. Conclusions: The present study provides valuable mitochondrial information for the classification of *P. macrochir* and new insights into the phylogenetic relationships of Polynemidae.

## 1. Introduction

Threadfins, specially the *Polydactylus* genus species, are easily identified due to their numerous, elongated, and threadlike pectoral fin rays [1]. These filamentous fin rays are necessary for threadfins inhabiting the sandy or muddy bottoms of turbid shallow waters to detect food, with both tactile and gustatory functions [2]. Threadfins belong to the family Polynemidae, which includes eight genera and 42 extant species [3]. The conservation management of Polynemidae is becoming increasingly vital for their commercial significance, with *Eleutheronema tetradactylum* (Shaw 1804) successfully adapted to the aquaculture industry [4,5], and some freshwater species to the aquarium trade [1].

Phylogenetic inference provides valuable insights into the origin and divergence history of morphological traits, taxonomic identification, and resource management and conservation. In recent years, the placement of Polynemidae among teleosts has varied according to different studies. Polynemidae had been previously considered related to Mugilidae, Sphyraenidae, Atherinidae, and Phallostethoidei and clustered into Mugiliformes [6]. A later study insisted that Polynemidae, Mugilidae, and Sphyraenidae should remain within the Perciformes [7]. The previous studies proposed that Polynemidae and Sciaenidae were sister groups [8,9,10], or aligned polynemids with Menidae [11], Menidae + Lactariidae [12], or Pleuronectiformes, respectively [13,14]. A study grouped Polynemidae with Menidae as the sister group of Sphyraenidae [15]. The recent study placed Polynemidae in the Order Polynemiformes [16]. As mentioned above, these different studies have advanced alternative hypotheses of the relationships for the family Polynemidae, but a consensual phylogenetic position is still lacking.

The mitochondrial genome, typically spanning 16–17k bp, generally consists of thirteen protein-coding genes, two ribosomal RNAs (rRNAs), twenty-two transfer RNAs (tRNAs), and two noncoding regions: the control region (CR) and the origin of L-strand replication (OL) [17]. Notably, mitochondrial DNA evolves at a faster rate and exhibits a remarkable degree of conservation in gene organization and a low recombination level [18]. In addition, the complete mitogenome has an advantage in providing comprehensive information when used for evolutionary analysis compared with a partial mitogenome gene. Thus, the complete mitogenome is used widely to study the phylogenetic relationships and species classification among teleosts [19,20,21]. To determine the precise phylogenetic inference of Polynemidae, there must be enough mitogenomic data. However, at present, there is a limited number of complete mitogenomes of Polynemidae species in the NCBI genebank.

Reconstructing the phylogenetic relationships between Polynemidae and its closely related species could help to infer the characteristics of their ancestors and follow the changing character states, which is important for the further exploration and utilization of threadfin germplasm resources. *Polydactylus macrochir* (Günther, 1867) is a large food and game fish which belongs to the family Polynemidae and is also named king threadfin. In this study, we sequenced the mitochondrial genome of *P. macrochir* and conducted genome characterization and phylogenetic analysis. This study will provide new insights regarding the classification of threadfins and will be helpful to resolve uncertainties of the phylogenetic position of Polynemidae.

## 2. Materials and Methods

### 2.1. Samples, DNA Extraction and Sequencing

One specimen of *P. macrochir* was purchased from the Fangchenggang local market, Guangxi, China. Muscle tissue was extracted and kept at 4 °C in a 2 mL centrifuge tube with 70% ethanol, a part of which was sent to Genepioneer Biotechnologies (Nanjing, China) for sequencing. Total genomic DNA was extracted using the MagPure Tissue DNA LQ Kit (Magen Biotechnology, Guangzhou, China). DNA purity was detected with 1.0% agarose gel. After that, the qualified library was sequenced using the Illumina Novaseq platform (San Diego, CA, USA), and the paired-end sequencing (PE) read length was 150 bp.

### 2.2. Genome Assembly and Annotation

fastp (v0.20.0, https://github.com/OpenGene/fastp, accessed on 20 October 2024) software was used to filter the original data and obtained clean data. To reduce the complexity of subsequent sequence assembly, the software bowtie2 v2.2.4 (http://bowtie-bio.sourceforge.net/bowtie2/index.shtml, accessed on 20 October 2024) was used to study the mitochondrial genome database built by the company, and the compared sequences were regarded as the mitochondrial genome sequences of the project samples. Then, the mt DNA sequence was assembled by SPAdes v3.10.1 software to obtain the seed sequence of the mitochondrial genome [22]. For a kmer iterative extend seed, if the result was a contig, the result was determined as a pseudo genome sequence. The sequence was aligned to a pseudo genome for genome correction. Annotations of the mitochondrial genome were performed in the Mitos2 web server [23] (parameters: E-value exponent = 5, maximum overlap = 100, ncRNA overlap = 100). The Mitos2 annotation results were compared with closely related species, and the final annotation results were obtained after manual correction. Mitochondrial gene structure maps were drawn using OGDRAW (https://chlorobox.mpimp-golm.mpg.de/OGDraw.html, accessed on 21 October 2024).

### 2.3. Codon Usage Analysis

Relative synonymous codon usage (RSCU) is thought to result from a combination of natural selection, mutation, and genetic drift; the numerical value is the ratio of the actual frequency of codon usage to the theoretical frequency of codon usage. A script wrote in Perl was used to filterUniq CDS (choose one of multiple copies of CDS) and perform the calculations.

### 2.4. Ka/Ks Value Analyses

To understand natural selection pressure in the evolution of the family Polynemidae, the homologous protein sequences between *P. macrochir* and other threadfins were obtained using BLASTN. Then, the shared protein-coding genes were aligned using MAFFT v7.427 [24]. The nonsynonymous (Ka) and synonymous (Ks) ratios (Ka/Ks) were calculated using KaKs_Calculator v2.0 [25].

### 2.5. Phylogenetic Analysis TimeTree Estimation

The mitochondrial genomes of 22 species were downloaded from GenBank (Table 1). The shared CDS was aligned using the MAFFT procedure. Maximum likelihood (ML): A maximum likelihood (ML) phylogenetic tree was conducted using RAxML v8.2.10 (https://cme.h-its.org/exelixis/software.html, accessed on 22 October 2024) (GTRGAMMA model) estimation with 1000 bootstrap replications. Bayesian inference (BI): The optimal nucleotide substitution model was calculated using jModelTestv2.1.10 (https://github.com/ddarriba/jmodeltest2, accessed on 22 October 2024), and then MrBayes v3.2.7a (http://nbisweden.github.io/MrBayes/, accessed on 22 October 2024) was used to establish a Bayesian inference (BI) phylogenetic tree; the parameters of MrBayes v3.2.7 software are based on the jModelTest v2.1.10 results. After inputting the sequence data, the constructed Bayesian inference topology (nwk format) was used as a baseline tree. Fossil records acquired from the TimeTree website (http://www.timetree.org, accessed on 24 October 2024) were used to calibrate the divergence times. We estimated the divergence times using PAML mcmctree (http://abacus.gene.ucl.ac.uk/software/paml.html, accessed on 24 October 2024).

## 3. Results

### 3.1. Mitogenomic Structure and Organization

**Figure 1:**
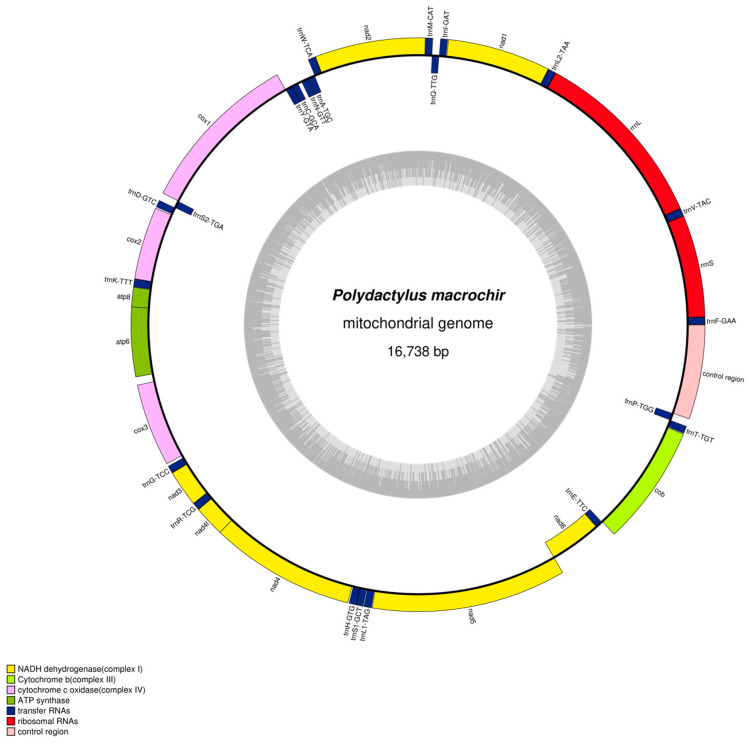
Mitochondrial genome map of *P. macrochir.*

In the present study, the mitogenome of *P. macrochir* (GenBank Accession No. PQ675801) was determined, with a total length of 16,738 bp. The complete length of the *P. macrochir* mitogenome is similar to those of the other sequenced Polynemidae species. It comprised thirteen protein-coding genes (PCGs), twenty-two transfer RNA genes (tRNAs), two ribosomal RNA genes (rRNAs), and one noncoding region (D-loop) (Figure 1). *NAD6* and eight tRNAs (*trnQ*, *trnA*, *trnN*, *trnC*, *trnY*, *trnS2*, *trnE*, and *trnP*) were located on the light (L) strand, while the remaining genes including twelve PCGs, two rRNAs, and fourteen tRNAs, were encoded by the heavy (H) strand. The *P. macrochir* mitogenome had six overlapping regions, with the longest overlapping region (10) detected between the *atp6* and *cox3* genes (Table 2). Nucleotide composition analysis showed that the *P. macrochir* mitogenome was AT-biased (54.15%). The PCGs, tRNAs, and rRNAs exhibited an AT content similar to that of the total mitogenomes. CR had the highest A + T content. All the AT skews in the mitogenome, tRNAs, rRNAs, and Dloop were positive, while the AT skew of the PCGs was negative. The GC skews of the mitogenome, PCGs, rRNAs, and Dloop were negative, while the GC skew of the tRNAs was positive. In addition, the GC skews for the complete mitogenomes and the PCGs were strongly negative, with values of −0.286 and −0.329, respectively (Table 3).

### 3.2. Protein-Coding Genes and Codon Usage

The total length of *P. macrochir* PCGs was 11,447 bp, accounting for 68.39% of the whole mitogenome. The largest PCG was *nad5*, with a length of 1839 bp, and the shortest PCG was *atp8*, with a length of 1839 bp. All the PCGs started with the regular codon ATG, except for *cox1*, which started with GTG. The termination codon varied across the PCGs. *nad6* terminated with the TAG stop codon. *nad2* terminated with the incomplete stop codon TA. *cox2*, *nad3*, and *nad4* used an incomplete T stop codon. The other PCGs shared the same complete stop codon TAA (Table 2).

**Figure 2:**
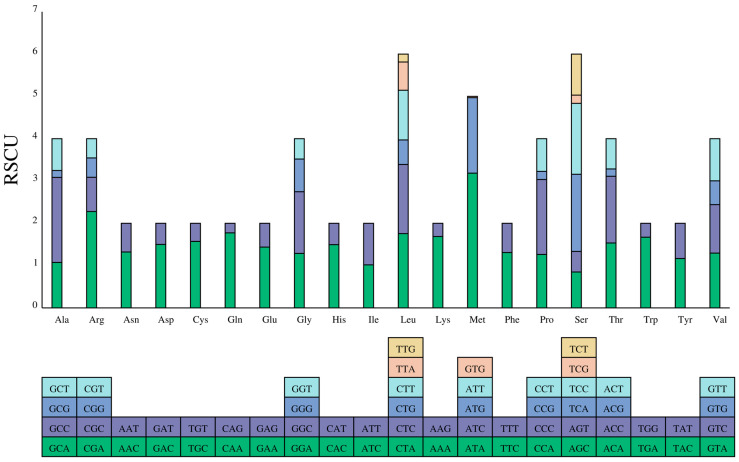
Relative synonymous codon usage (RSCU) in the mitogenome of *P. macrochir.* (The *y*-axis represents the usage frequency of the corresponding amino acid codons in 13 PCGs. The different colors represent different codons in the amino acid).

The relative synonymous codon usage (RSCU) values of the PCGs are revealed in Figure 2. There were 30 codons with RSCU  >  1 (Supplementary Materials). The number of codons ending with the A base was fourteen, with one codon ending with U. Similarly, there were fourteen codons ending with C and one codon ending with G. The results also showed that serine and leucine were encoded by six codons with greater codon abundance compared to the other amino acids.

### 3.3. Ka/Ks Value Analyses

**Figure 3:**
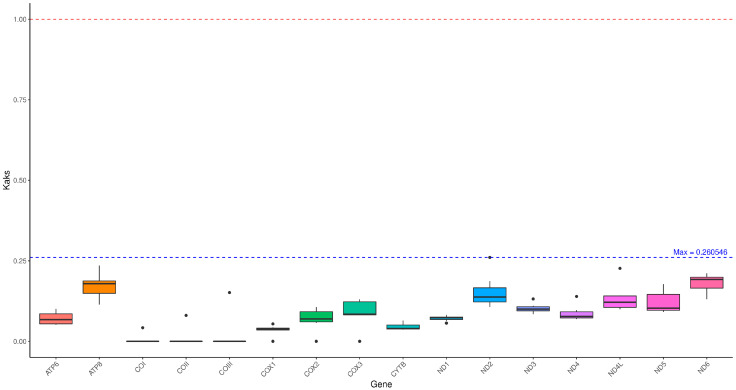
Ka/Ks values for 13 PCGs of *P. macrochir* compared to other threadfins chose in this study.

To evaluate the selective pressures of the PCGs, we detected nonsynonymous (Ka) and synonymous (Ks) substitution rates. It was shown that the ka/ks ratios of all the PCGs were less than one. The Ka/Ks ratio was the lowest in *cox1* (Figure 3, Supplementary Materials).

### 3.4. Phylogenetic Analysis and TimeTree Estimation

**Figure 4:**
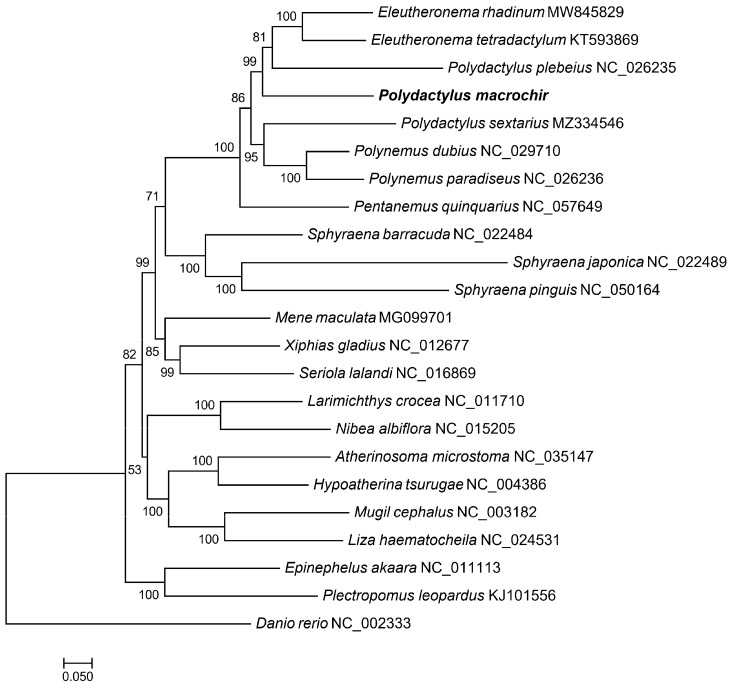
ML phylogenetic trees based on the nucleotide datasets for 13 PCGs from the mitogenomes of 23 species (Polynemidae, Sphyraenidae, Xiphiidae, Carangidae, Menidae, Sciaenidae, Atherinidae, Serranidae, Mugilidae, and Danionidae). *P. macrochir* was sequenced in this study. The bootstrap values are overlaid with each node.

**Figure 5:**
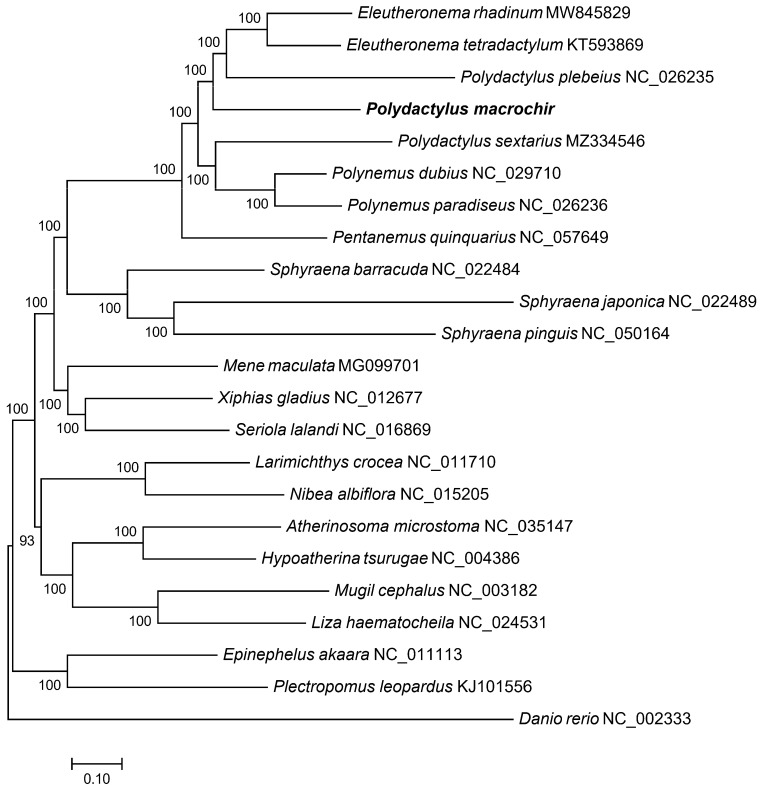
BI phylogenetic trees based on the nucleotide datasets for 13 PCGs from the mitogenomes of 23 species (Polynemidae, Sphyraenidae, Xiphiidae, Carangidae, Menidae, Sciaenidae, Atherinidae, Serranidae, Mugilidae, and Danionidae). *P. macrochir* was sequenced in this study. The bootstrap values are overlaid with each node.

**Figure 6:**
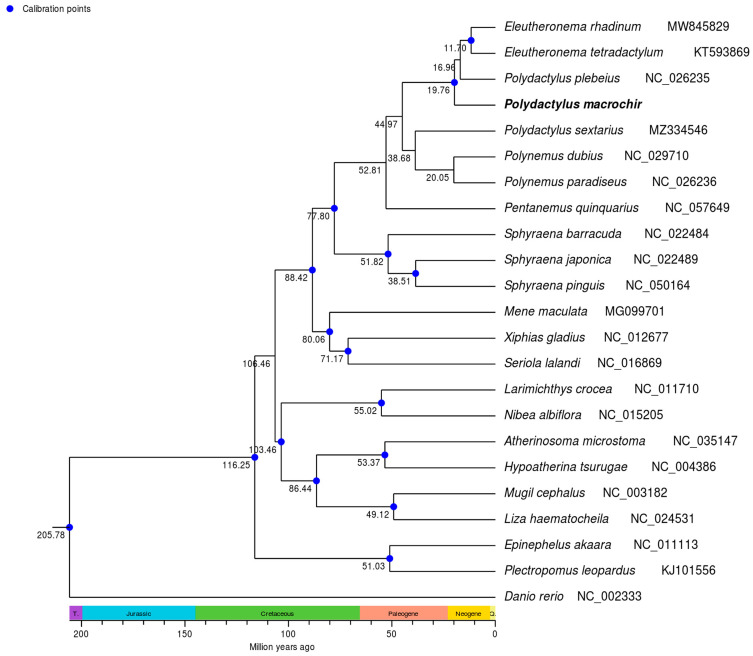
Divergence time estimation of threadfins and other fish species (Polynemidae, Sphyraenidae, Xiphiidae, Carangidae, Menidae, Sciaenidae, Atherinidae, Serranidae, Mugilidae, and Danionidae). *P. macrochir* was sequenced in this study. Numbers at nodes indicate estimated age.

To find the position of *P. macrochir* among the 22 other species, a phylogenetic tree was constructed using maximum likelihood (ML) and Bayesian inference (BI) based on the coding sequence. It is shown that the ML trees and the BI trees share a similar topological structure (Figure 4 and Figure 5). As an outgroup, *Danio rerio* was distinct from the other fish species. The remaining fish species were grouped into two major clades. Eight polynemid species formed a well-supported monophyletic cluster. *P. macrochir* and the monophyletic cluster consists of *Eleutheronema tetradactylum* and *Polydactylus plebeius,* forming a sister group. Polynemidae formed a sister cluster with the clade of Sphyraenidae with a high bootstrap support value (100%). Then, (Polynemidae + Sphyraenidae) and (Menidae + Xiphiidae + Carangidae) were a sister lineage. The other representative species from the different families display distinct monophyletic clustering patterns. According to TimeTree analyses, the most recent common ancestor (MRCA) of Polynemidae was traced back to about 52.81 million years ago (MYA), while six species within Polynemidae diverged from 11.70 MYA to 20.05 MYA (Figure 6).

## 4. Discussion

The mitochondrial genome of *P. macrochir* has a total length of 16,738 bp, which is within the range of those of the other seven sequenced mitogenomes of threadfins. A typical set of 37 genes like that of other vertebrates [26], containing 13 PCGs, 22 tRNAs, 2 rRNAs, and 1 noncoding CR, was detected in this study. In terms of gene arrangement, *P. macrochir* was consistent with the other sequenced related species [16]. Both of these results indicate that *P. macrochir* was conserved. The nucleotide composition of the *P. macrochir* mitogenome was rich in A  +  T (54.15%), with a slightly positive AT skew (0.049), while the GC skew was moderately negative (−0.286). The overlapping and intergenic regions, which are commonly observed in other species, were both present in the mitogenome of *P. macrochir.* The length of the overlapping and intergenic regions between adjacent genes varied with the species lineage. All the PCGs started with typical ATN codons, except for *cox1*, initiated by GTG. In *P. macrochir*, TAA was the most common codon used as a stop codon, while the TAG terminating codon was used once, with the remaining incomplete T and TA codons. Atypical initiation codons and incomplete terminating codons have also been reported in the mitogenomes of other fish species [27].

As we know, the RSCU usually reflects the preference for codon usage [28]. In *P. macrochir*, the PCGs were more prone to use A and C than T and G at the third codon, which is different from Epinephelidae [29]. This bias in the usage of codons may provide hints to understand the evolutionary history of *P. macrochir.*

The analysis of nonsynonymous (Ka) and synonymous (Ks) substitution rates within miogenome PCGs provides evidence for studying natural selection and local adaption [30]. In this study, the Ka/Ks ratio of all the PCGs was much less than 1, indicating strong negative selection among the selected species. Mitochondrial PCGs play a crucial role in oxygen utilization and energy metabolism, which are vital for the survival and development of an organism. The low Ka/Ks ratio of all the PCGs ensure their functional conservation, reflecting the evolutionary strategy for ecological niche adaptation.

The previous studies did not resolve the uncertainties of the Polynemidae phylogenetic position. In this study, we reconstructed a phylogenetic tree for *P. macrochir and* 22 other species based on 13 PCGs from the mitochondrial genome. The same topologies were generated by both BI and ML analyses. *P. macrochir* and another seven species of Polynemidae were clustered into a clade with high nodal support values, validating the monophyly of the family Polynemidae. Within Polynemidae, *P. macrochir* and the other *Polydactylus* species cannot form a monophyletic group. The present results emphasize the need to revise the genus *Polydactylus* in accordance with the monophyletic principle. Therefore, more mitogenomes of the genus *Polydactylus* need to be sequenced to reconstruct the true phylogenetic tree. It was also shown that Polynemidae and Sphyraenidae are sister groups. Furthermore, the sister group relationships between (Polynemidae + Sphyraenidae) and (Menidae + Xiphiidae + Carangidae) were robustly supported. These results conflicted with the earlier studies which proposed that polynemids are closer to Mugilidae, Sphyraenidae, Atherinidae, and Phallostethoidei; Mugilidae and Sphyraenidae; or Sciaenidae according to different phenotypic analyses [6,7,8,9,10]. Morphological convergence was a primary source of error in phylogenetic reconstruction, and it was also difficult to assess trait homology among the taxa. Morphology inference based on different data partitions usually generated different topologies [31], which also happened in reconstructing the phylogenetic placement of Polynemidae. The alternative hypotheses for the phylogenetic placement of Polynemidae based on molecular analyses, such as Polynemidae grouped with Menidae and Lactariidae [12], Polynemidae and Menidae as a sister lineage [11], and Polynemidae + Menidae as a sister group of Sphyraenidae [15], are also in conflict with the present study. These phylogenetic analyses based on a single gene or combination of some genes were not powerful enough to build robust phylogenetic inference when compared with mitogenome-based analysis. Recently, the sister group relationship between Polynemidae and Pleuronectiformes was supported by molecular and morphological data [13,14]. In this study, the relationship between Polynemidae and Pleuronectiformes was not involved, which should be resolved by future research. According to the present results, we tend to locate Polynemidae in Perciformes and do not support setting the independent Order Polynemiformes [16]. Polynemidae and Sphyraenidae clustered together as a monophyletic group might form a new suborder of Perciformes, but we are in need of more evidence. The estimation of divergence time displayed that Polynemidae and Sphyraenidae diverged from the most recent common ancestor in the late Cretaceous at 77.80 MYA. Threadfins may originate in the Paleogene at 52.81 MYA. Some species of Polynemida may have undergone accelerated diversification during the Neogene period. This enhances our understanding of the phylogenetic relationships and speciation of Polynemidae. In future studies, improved taxon sampling and genome data are necessary to resolve phylogenetic incongruence, and what is important is the integration of molecular and morphology datasets to estimate the full evolutionary history of Polynemidae.

## 5. Conclusions

In this study, the complete mitogenome of *P. macrochir* was sequenced and analyzed. The mitochondrial genome had a total size of 16,738 bp and had conserved gene contents and arrangement. The PCGs tended to use A and C rather than T and G at the third codon. All of the PCGs underwent purifying selection. Phylogenetic analysis revealed that Polynemidae was clustered into a monophyletic group, being a sister group to Sphyraenidae. Threadfins may have originated in the Paleogene at 52.81 MYA. Some species of Polynemida may have undergone accelerated diversification during the Neogene period. These results provide valuable information for future studies on the phylogenetic relationships and speciation of Polynemidae.

## Figures and Tables

**Table 1:** Species attribution and accession number.

Order	Family	Genus	Species	Size (bp)	Accession No.
Perciformes	Polynemidae	*Eleutheronema*	*Eleutheronema rhadinum*	16,718	MW845829.1
			*Eleutheronema tetradactylum*	16,491	KT593869.1
		*Pentanemus*	*Pentanemus quinquarius*	16,708	NC_057649.1
		*Polydactylus*	*Polydactylus sextarius*	16,841	MZ334546.1
			*Polydactylus plebeius*	16,765	NC_026235.1
		*Polynemus*	*Polynemus paradiseus*	16,710	NC_026236.1
			*Polynemus dubius*	16,555	NC_029710.1
	Sphyraenidae	*Sphyraena*	*Sphyraena pinguis*	16,620	NC_050164.1
			*Sphyraena* *barracuda*	16,707	NC_022484.1
			*Sphyraena japonica*	16,760	NC_022489.1
	Xiphiidae	*Xiphias*	*Xiphias gladius*	16,520	NC_012677.1
	Carangidae	*Seriola*	*Seriola lalandi*	16,532	NC_016869.1
	Menidae	*Mene*	*Mene maculata*	16,733	MG099701.1
	Sciaenidae	*Larimichthys*	*Larimichthys crocea*	16,466	NC_011710.1
		*Nibea*	*Nibea albiflora*	16,499	NC_015205.1
Atheriniformes	Atherinidae	*Atherinosoma*	*Atherinosoma microstoma*	16,573	NC_035147.1
		*Hypoatherina*	*Hypoatherina tsurugae*	16,566	NC_004386.1
	Serranidae	*Epinephelus*	*Epinephelus akaara*	16,795	NC_011113.1
		*Plectropomus*	*Plectropomus leopardus*	16,754	KJ101556.1
Mugiliformes	Mugilidae	*Mugil*	*Mugil cephalus*	16,685	NC_003182.1
		*Planiliza*	*Liza haematocheila*	16,822	NC_024531.1
Cypriniformes	Danionidae	*Danio*	*Danio rerio*	16,596	NC_002333.2

**Table 2:** General features of mitogenome of *P. macrochir.*

		Position				Codon	
Gene	Stand	From	To	Size	Intergenic Length	Start	Stop
*trnF*	H	1	70	70	0		
*rrnS*	H	71	1037	967	0		
*trnV*	H	1038	1110	73	0		
*rrnL*	H	1111	2853	1743	0		
*trnL2*	H	2854	2927	74	0		
*nad1*	H	2928	3902	975	0	ATG	TAA
*trnI*	H	3907	3976	70	4		
*trnQ*	L	3976	4046	71	−1		
*trnM*	H	4046	4114	69	−1		
*nad2*	H	4115	5160	1046	0	ATG	TA-
*trnW*	H	5161	5231	71	0		
*trnA*	L	5233	5301	69	1		
*trnN*	L	5303	5375	73	1		
*trnC*	L	5414	5479	66	38		
*trnY*	L	5480	5549	70	0		
*cox1*	H	5551	7101	1551	1	GTG	TAA
*trnS2*	L	7102	7173	72	0		
*trnD*	H	7174	7242	69	0		
*cox2*	H	7252	7942	691	9	ATG	T--
*trnK*	H	7943	8016	74	0		
*atp8*	H	8018	8206	189	1	ATG	TAA
*atp6*	H	8176	8859	684	−31	ATG	TAA
*cox3*	H	8933	9718	786	73	ATG	TAA
*trnG*	H	9742	9813	72	23		
*nad3*	H	9814	10,162	349	0	ATG	T--
*trnR*	H	10,163	10,231	69	0		
*nad4l*	H	10,232	10,528	297	0	ATG	TAA
*nad4*	H	10,522	11,896	1375	−7	ATG	T--
*trnH*	H	11,906	11,974	69	9		
*trnS1*	H	11,975	12,043	69	0		
*trnL1*	H	12,048	12,120	73	4		
*nad5*	H	12,124	13,962	1839	3	ATG	TAA
*nad6*	L	13,959	14,480	522	−4	ATG	TAG
*trnE*	L	14,482	14,551	70	1		
*cob*	H	14,556	15698	1143	4	ATG	TAA
*trnT*	H	15,703	15,774	72	4		
*trnP*	L	15,774	15,845	72	−1		
D-loop	H	15,846	16,738	893	0		

**Table 3:** Nucleotide composition and skewness values of *P. macrochir* mitogenome.

Polydactylus_Macrochir	Size(bp)	A%	T%	G%	C%	A + T%	G + C%	AT-Skew	GC-Skew
Mitogenome	16,738	28.4	25.75	16.36	29.49	54.15	45.85	0.049	−0.286
PCGs	11,447	25.6	27.71	15.65	31.03	53.32	46.68	−0.039	−0.329
tRNAs	1557	27.49	26.59	23.83	22.09	54.08	45.92	0.017	0.038
rRNAs	2710	31.51	22.77	20.92	24.8	54.28	45.72	0.161	−0.085
Dloop	893	35.72	31.02	14.22	19.04	66.74	33.26	0.07	−0.145

## Data Availability

The data presented in this study are available in NCBI GenBank (Accession number: PQ675801).

## Supplementary Materials

The following supporting information can be downloaded at: https://www.mdpi.com/article/10.3390/genes16010088/s1, Table S1: ka/ks value list and relative synonymous codon usage (RSCU) of *P. macrochir*.
